# Effect of a Probiotic Beverage Enriched with Cricket Proteins on the Gut Microbiota: Composition of Gut and Correlation with Nutritional Parameters

**DOI:** 10.3390/foods13020204

**Published:** 2024-01-09

**Authors:** Chaima Dridi, Mathieu Millette, Stephane Salmieri, Blanca R. Aguilar Uscanga, Sebastien Lacroix, Tommaso Venneri, Elham Sarmast, Zahra Allahdad, Vincenzo Di Marzo, Cristoforo Silvestri, Monique Lacroix

**Affiliations:** 1 Research Laboratories in Sciences, Applied to Food (RESALA), Canadian Irradiation Centre (CIC), INRS Armand-Frappier Health Biotechnology Research Centre, Laval, QC H7V 1B7, Canadamathieu.millette@kerry.com (M.M.);; 2 Institute of Nutrition and Functional Foods (INAF), Université Laval, Quebec, QC G1V 0A6, Canada; 3 Bio-K+, a Kerry Company, Preclinical Research Division, Laval, QC H7V 4B3, Canada; 4 Research Laboratory of Industrial Microbiology, Centro Universitario de Ciencias Exactas e Ingenierías, Universidad de Guadalajara, Guadalajara 44430, Mexico; blanca.aguilar@academicos.udg.mx; 5 Faculty of Agriculture and Food Sciences (FSAA), Université Laval, Quebec, QC G1V 0A6, Canada; cristoforo.silvestri@criucpq.ulaval.ca; 6 Joint International Research Unit on Chemical and Biomolecular Research on the Microbiomeand Its Impact on Metabolic Health and Nutrition (UMI-MicroMeNu), Université Laval, Quebec, QC G1V 0A6, Canada; 7 Heart and Lung Institute Research Centre (IUCPQ), Université Laval, Quebec, QC G1V 0A6, Canada; 8 Nutrition, Health and Society (NUTRISS) Centre, Department of Medicine, Faculty of Medicine, Université Laval, Quebec, QC G1V 0A6, Canada

**Keywords:** cricket protein hydrolysates, probiotics, gut microbiota, nutritional quality, metagenomics

## Abstract

The health and balance of the gut microbiota are known to be linked to diet composition and source, with fermented products and dietary proteins potentially providing an exceptional advantage for the gut. The purpose of this study was to evaluate the effect of protein hydrolysis, using a probiotic beverage enriched with either cricket protein (CP) or cricket protein hydrolysates (CP.Hs), on the composition of the gut microbiota of rats. Taxonomic characterization of the gut microbiota in fecal samples was carried out after a 14-day nutritional study to identify modifications induced by a CP- and CP.H-enriched fermented probiotic product. The results showed no significant differences (*p* > 0.05) in the diversity and richness of the gut microbiota among the groups fed with casein (positive control), CP-enriched, and fermented CP.H-enriched probiotic beverages; however, the overall composition of the microbiota was altered, with significant modifications in the relative abundance of several bacterial families and genera. In addition, fermented CP.H-enriched probiotic beverages could be related to the decrease in the number of potential pathogens such as *Enterococcaceae*. The association of gut microbiota with the nutritional parameters was determined and the results showed that digestibility and the protein efficiency ratio (PER) were highly associated with the abundance of several taxa.

## 1. Introduction

Intestinal microbiota is very important for health, playing a key role in proper digestion, the functioning of the immune system, and other aspects of health [1]. On the other hand, an imbalanced gut microbiota can contribute to the development of various health conditions such as elevated circulating blood lipids, obesity, and high blood sugar [2]. Several factors can affect the balance of gut microbial communities, including health status, age, and, most importantly, diet [3].

With this in mind, probiotic-fortified food products are in high demand, as they have the potential to alter the composition of gut microbiota while resulting in beneficial health effects [4,5]. They have been shown to lead to reduction of gut inflammation [6], prevention of antibiotic-associated diarrhea (AAD), and improvement of symptoms associated with irritable bowel syndrome (IBS) [7].

Dietary protein has significant effects on the gut microbiota in a source-dependent manner, with increased prevalence of pathogenic bacteria being associated with higher levels of undigested protein [8,9,10], though several open questions regarding the interaction between the gut microbiota and dietary protein remain, not the least of which is the impact of protein digestibility [11]. Further, the absorption of protein could affect postprandial energy metabolism [8]. This effect is conducted by the gut microbiota by converting the dietary proteins into other metabolically active compounds such as short-chain fatty acids, branched chain fatty acids, and different nitrogen-containing compounds [8,12]. 

In recent years, insects have come to be considered one of the most interesting protein sources, given their amino-acid composition, levels of other nutrients, and low environmental impact compared to traditional farm animals [13]. A growing body of research suggests that the consumption of insects could have a positive impact on human health, stimulating the growth of the intestinal microbiota, improving immune function, and decreasing inflammatory factors [14,15]. On the other hand, insects have been shown to be rich in antimicrobial peptides [16]. Insects are also a source of fiber, namely chitin, which can lead to poor digestibility and reduce essential amino-acid (EAA) bioavailability [17]. Therefore, the partial or total elimination of chitin could be a good solution for improving the nutritional aspect of insect proteins [18]. Some physical processes, such as irradiation and ultrasound, and biological treatments, such as enzymatic hydrolysis using protease enzymes and fermentation by lactic acid bacteria, have been successfully applied to greatly improve the digestibility and the nutritional and functional properties of insect proteins and to decrease their allergenicity [19,20,21,22,23].

This work is a continuation of a previous 14-day in vivo rat study, in which the nutritional quality of a probiotic beverage enriched with either cricket protein in its native (CP) or hydrolysed (CP.H) form was investigated in order to determine the effect of hydrolysis pre-treatment on nutritional parameters such as the protein efficiency ratio (PER), the net protein efficiency ratio (NPR), true digestibility (TD), and apparent digestibility (AD) [24]. Cricket protein was utilized because its amino acid composition and scores meet the requirements of the World Health Organization (WHO), with an EAA score of 44.5% (>36% required) [24]. The results of the study indicated that cricket proteins benefit from a complete amino-acid profile rich in growth-including methionine and cysteine [24]. Moreover, nutritional improvements of NPR, PER, and AD (similar to that of casein) were observed in protein hydrolysates compared to whole proteins. Here, we evaluate the effect of the above-described 14-day protocol on the composition of the gut microbiota and the association of bacterial taxa with diet nutritional parameters.

## 2. Materials and Methods

### 2.1. Cricket Protein Hydrolysates Preparation

The fermented and the non-fermented beverages used in this study were produced by Bio-K Plus International Inc., a Kerry company (Laval, QC, Canada). Organic cricket flour (60% protein content) was produced by Nexxus Foods (Montreal, QC, Canada). The cricket protein hydrolysates were prepared in two steps, according to a previous study carried out in our laboratory [25]: the first step consisted of pretreating a cricket protein suspension (40% *w*/*v*) by ultrasound (US) using a QSonica Q500 sonicator (model FB-505; Fisher Scientific, Ottawa, ON, Canada). The parameters of the sonicator were programmed as follows: power of 500 W, frequency of 20 kHz, amplitude of 60%, and treatment duration of 15 min in pulsed mode. Then, the cricket suspension was submitted to an enzymatic hydrolysis using Alcalase^®^ 2.4 L FG (Novozymes A/S, Bagsvaerd, Denmark), according to the following conditions: enzyme/substrate (E:S) ratio of 1:10 (*w*/*w*), reaction time of 180 min, hydrolysis temperature of 55 °C, and pH 8.0. Then, the suspension was heated to 95 °C for 10 min for enzyme inactivation. Afterwards, the suspension was cooled down at room temperature and centrifuged at 13,000× *g* for 20 min. The supernatant was lyophilized (Labconco Freezone^®^ 2.5 L, model 7670521, Fisher Scientific) to form protein hydrolysates (CP.Hs) used for the enrichment of fermented beverages. 

### 2.2. Preparation of Beverages

A commercial Bio-K+^®^ blueberry fermented beverage, in powdered form, was used for enrichment. The powder was weighed and hydrated with filtered water before being enriched with cricket powder having a total protein content of 13% and the proteins were pre-treated with selected processes, as described previously [24,25,26]. Other beverages non-enriched (with 3% of protein) and non-fermented were produced and used as controls for comparison. The beverage was then pasteurized at 90 ± 2 °C for 60 s, then cooled to 37 °C. Individual probiotic bacteria (*Lactobacillus acidophilus* CL1285, *Lacticaseibacillus casei* LBC80R, and *Lacticaseibacillus rhamnosus* CLR2) were kept frozen at −80 °C in MRS broth containing glycerol (20% *w*/*v*). The starter culture was prepared by thawing each individual strain, then inoculating the bacteria in increasing volumes of MRS broth. These cultures were then placed at 37 °C for 24 h three consecutive times. Finally, bacteria were mixed, and this represented the starter culture used to ferment the protein-enriched beverage. The beverage was inoculated with Bio-K+™ starter culture at 10^8^ CFU/mL, packaged in bottles containing 98 g of product, aluminum-sealed, incubated at 37 ± 1 °C for 14 ± 2 h, and cooled to 4 °C. Another beverage was prepared using the same protocol, but without addition of the probiotic bacteria. This preparation served as control. The beverages were then freeze-dried and milled to pass through a 20-mesh sieve prior to preparation of the diets. The total weight of the freeze-dried products was 1.1 kg each, with a 49.5% protein content.

### 2.3. Preparation of Experimental Diets and Animal-Study Design

Briefly, four diets (in pellet form) were produced and evaluated: CP-based and CP.H-based supplemented probiotic beverage diets, casein-containing diets, and protein-free diets. The formulations were prepared according to an official method of analysis (AOAC 960.48) [27]. The percentage of proteins was adjusted to 10% for all the formulations except the protein-free diet, which served as a negative control. Casein was used as a reference protein (positive control). The lyophilized fermented beverages’ CP and CP.H were used as protein sources. Soybean oil, vitamins, minerals, cellulose, sucrose, and starch were also added to the formulations and their amounts were calculated to obtain equal calorie counts for all diets [24]. The dose of probiotics used to ferment the beverages was a standard dose of the combination of 3 probiotic strains that allowed for an adequate fermentation profile and appropriate organoleptic properties of the fermented beverages (from the manufacturer’s protocol of Bio-K+, a Kerry company). 

For the animal study, the experiment was conducted according to prior approval by the National Experimental Biology Laboratory (LNBE) and the Institutional Animal Care Committee (CIPA) of the INRS–Armand–Frappier Health and Biotechnology Research Centre, in accordance with the principles of the Canadian Council on Animal Care (CCAC), by using the CIPA no. 1809-04 protocol [24]. Male Wistar rats, 20–23 days old, were distributed in 4 groups of 7 rats and housed in separate cages. Cycles of 12 hours of light–dark and a temperature of 20 ± 0.5 °C were fixed throughout the experiment period. For the first 5 days (the acclimation phase), the rats received a standard diet. In the subsequent 14 days, the rats were fed ad libitum with the protein-free (*n* = 4), casein (*n* = 5), CP (*n* = 5), or CP.H (*n* = 5) experimental diets. The survival rate of the experimental animals was 100%.

In terms of study limitations, this work was carried out in a rodent model; thus, it assessed effects on a rodent microbiota, which is different from that of humans. Thus, the results may not all translate to humans. Furthermore, the duration of the study was only 14 days, and a longer protocol may have resulted in different observations with respect to the adaptation of bacteria to the diets. Also, we did not vary the protein concentration, which could also reduce the impact on the results. Finally, the experimental design was limited to the effect of fermented, probiotic, cricket protein-enriched beverages compared to a non-fermented casein-based diet (without probiotic), used as a positive control for the in vivo test.

### 2.4. rDNA Sequencing and Metagenomic Analysis

At the end of 14 days of experiment, feces were collected from the colons of the rats and stored in a sterile plastic tube. DNA from fecal samples was extracted using the Qiagen DNeasy PowerSoil kit. The DNA concentrations of the extracts were measured fluorometrically with the Quant-iT PicoGreen dsDNA kit (Thermo Fisher Scientific, Waltham, MA, USA), and the DNA samples were stored at −20 °C until 16S rDNA library preparation was completed according to the Illumina “Preparing 16S Ribosomal RNA Gene Amplicons for the Illumina MiSeq System” protocol. Briefly, 15 ng of DNA was used as the template, and the V3–V4 region of the 16S rRNA gene was amplified by PCR using the following primers: 16S Amplicon PCR forward primer = 5′-TCGTCGGCAGCGTCAGATGTGTATAAGAGACAGCCTACGGGNGGCWGCAG-3′, 16S Amplicon PCR reverse primer = 5′-GTCTCGTGGGCTCGGAGATGTGTATAAGAGACAGGACTACHVGGGTATCTAATCC-3′ followed by a second PCR reaction to introduce indices (Nextera XT Index; Illumina, San Diego, CA, USA). The 16S metagenomic libraries were eluted in 30 μL of nuclease-free water and 1 μL was qualified with a Bioanalyser DNA 1000 chip (Agilent Technologies, Santa Clara, CA, USA) to verify the amplicon size (expected size ∼600 bp) and then quantified with a Qubit (Thermo Fisher Scientific, Waltham, MA, USA). The libraries were then normalized and pooled to 2 nM, denatured, diluted to a final concentration of 10 pM, and supplemented with 5% PhiX control (Illumina). Sequencing (2 × 300 bp paired-end) was performed using the MiSeq reagent kit V3 (600 cycles) on an Illumina MiSeq system (Illumina, San Diego, CA, USA). Sequencing reads were generated in less than 65 h. Image analysis and base calling were carried out directly on the MiSeq. The preprocessing of obtained sequences and bacterial taxa assignation was performed according to the Dada2 pipeline (version 1.10.1) using the Ribosomal Database Project (RDP release 11) reference database [28]. Analyses were then conducted on sequence counts normalized by cumulative-sum scaling (CSS) (MetagenomeSeq R package) [29]. CSS divides sequence counts by the median sequence counts of each sample [30]. CSS-normalized counts are thus expressed in relation to the entire bacterial composition of each sample and are viewed as being more appropriate than total-sum scaling [31].

In order to determine if the nutritional parameters [24] related to different diets (see above) after feeding the rats for 14 days correlated with alterations in microbial composition, we performed a Spearman’s rank-order correlation.

### 2.5. Statistical Analysis

The gut microbiota characteristics, the potential relationship between the diet groups, and the microbial community structure were examined via PCA and PCoA, performed using Bray–Curtis dissimilarity indices of beta-diversity and permutational multivariate analysis of variance (PERMANOVA) (VeganR package) [32]. Two-way ANOVA (taxa abundance ~ Genotype*Diet), followed by the Tukey HSD post hoc test, was conducted to evaluate the influence of the diet group and the protein type on gut microbial taxa. The results were considered statistically significant at *p* < 0.05 or FDR-adjusted *p* < 0.1. Analyses were performed with R software, version 3.4.3. [29].

## 3. Results and Discussion

### 3.1. Gut Microbiota: Diet Effect 

#### 3.1.1. Diet Effect on Gut Microbiota: Comparison of PCA and PCoA Methods

**Figure 1:**
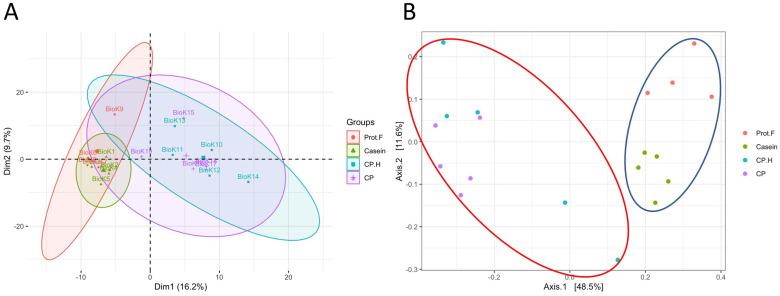
Comparison between the gut microbiota of rats according to diet and protein-type feeding. (**A**) Principal component analysis (PCA), (**B**) principal coordinate analysis (PCoA) of fecal microbiota from feed with different diets. Permutational multivariate analysis of variance (PERMANOVA) confirmed that the microbiota from rats fed with cricket protein-containing fermented beverage-based diets (red ellipse) were different from those fed control diets (blue ellipse). Each point represents one biological sample. Prot.F: protein free-based control diet; casein: casein-based control diet; CP.H: cricket hydrolysates-enriched fermented beverage diet; CP: whole-cricket protein-enriched fermented beverage diet.

We analyzed whether protein-free, casein-, CP-based, and CP.H-based fermented beverage diets modified the gut microbiota of rats after 14 days of ad libitum feeding. Given the rapidity at which the gut microbiota responds to dietary interventions [33], we hypothesized that our protocol would result in demonstrable differences between the experimental groups. Principle component analysis (PCA) suggested that the casein-based diet did not significantly alter the gut microbiota compared to the protein free-based diet, as both groups clustered together, while the fermented CP-based and CP.H-based diets clustered mostly independently from the control diets (Figure 1A). Subsequent principal coordinate analysis (PCoA) followed by permutational multivariate analysis of variance (PERMANOVA) confirmed that the fecal microbiota of rats fed the protein free-based and casein-based diets were not significantly different from one another (*p* > 0.05); however, the fermented CP.H and CP diets were statistically different from the casein-based diet (*p* = 0.024 and *p* = 0.024, respectively) and the protein-free diet (*p* = 0.049 and *p* = 0.024, respectively) (Figure 1B).

#### 3.1.2. Diet Effect on Microbial Diversity: Comparison of Shannon Alpha-Diversity Index and Firmicutes-to-Bacteroidetes Ratio

**Figure 2:**
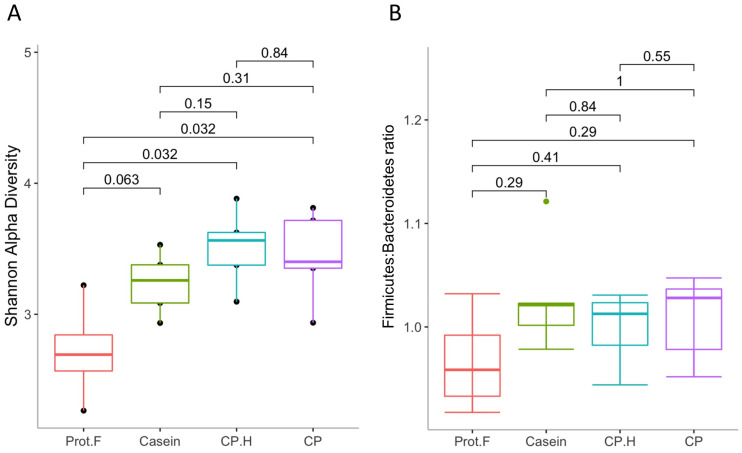
(**A**) Shannon alpha-diversity index, evaluating gut microbiota richness and evenness and (**B**) *Firmicutes*-o-*Bacteroidetes* ratio. Prot.F: protein free-based control diet; casein: casein-based control diet; CP.H: cricket hydrolysates-enriched fermented beverage; CP: whole-cricket protein-enriched fermented beverage.

The effect of the diet group on microbial diversity within the gastrointestinal tract was determined using the Shannon alpha-diversity index (Figure 2A). Supplementation with casein showed a trend for increased alpha diversity compared to the protein-free diet (*p* = 0.063); however, both the CP.H-based and CP-based diets had significantly increased alpha diversity at the end of the protocol. No difference in alpha diversity was observed between casein-, CP.H-based, and C.P-based diets, indicating that the alpha diversity of gut microbiota increased due to the diverse nutrients, such as the amino acids provided by protein-based diets that serve to provide a variation of substrates [34,35]. These data contrast with the general observation that increased protein consumption is associated with decreased bacterial diversity [36,37]; however, none of these studies compared the effects to a protein-free diet, as we have here. None of the diets showed any differences in the *Firmicutes*-to-*Bacteroidetes* ratio (F:B ratio) (Figure 2B). However, we noted a non-statistically significant trend for a decreased F:B ratio in the group fed the protein free-based diet compared to groups fed the protein-based diets (casein, fermented CP.H, and fermented CP). While the F:B ratio has been thought to correlate with obesity and other diseases [38,39], recent evidence has cast doubt on this with respect to obesity [40,41]; the protein-based diet used in this study did not cause obesity, as the rats in our study were all of normal weight for their age [24]. However, while the animals fed with protein did not show obese characteristics, animals fed the protein free-based diet showed a loss of weight after 14 days (from 103.3 ± 3.1 g to 78.8 ± 1.9 g). The results of this research are in a good agreement with those of Stull et al. (2018) [16] in humans and Jarett et al. (2019) [42] in dogs, in which cricket consumption did not alter alpha and beta diversity when compared to other proteins used in the studies. Increased alpha diversity is generally associated with weight loss [43]; however, again, we point out that our control diet reflects an extreme, in that it is protein-free and that most studies assessing alpha diversity and weight loss focus on experimental protocols that result in overweight/obesity or participants who are overweight/obese. 

#### 3.1.3. Diet Effect on the Relative Abundance of Gut Microbiota at Phylum and Family Levels

**Figure 3:**
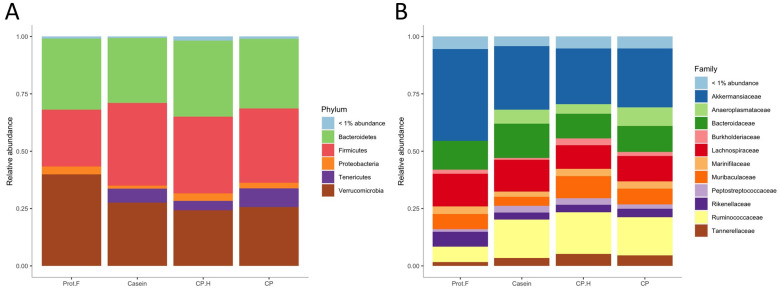
(**A**) Relative abundance of gut microbiota at the phylum, (**B**) family level. Prot.F: protein free-based diet; casein: casein-based control diet; CP.H: cricket hydrolysates-enriched fermented beverage; CP: whole-cricket powder-enriched fermented beverage.

The relative abundance of fecal bacteria from rats fed the different diets is presented in Figure 3. At the phylum level (Figure 3A), the most predominant phyla for the four groups were *Verrucomicrobia*, *Bacteroidetes*, *Firmicutes*, and *Proteobacteria*. Further, an additional phylum (*Tenericutes*) was significantly increased in all protein-containing groups, compared to the protein-free group (casein vs. protein-free; *p* = 6.7 × 10^−8^, CP.H vs. protein-free; *p* = 7.5 × 10^−7^, CP vs. protein-free; *p* = 1.9 × 10^−7^). It may be that members of the *Tenericutes* phylum are beneficial for the integrity of the intestine. It has also been found to be decreased within inflammatory bowel disease patients [44]. 

**Figure 4:**
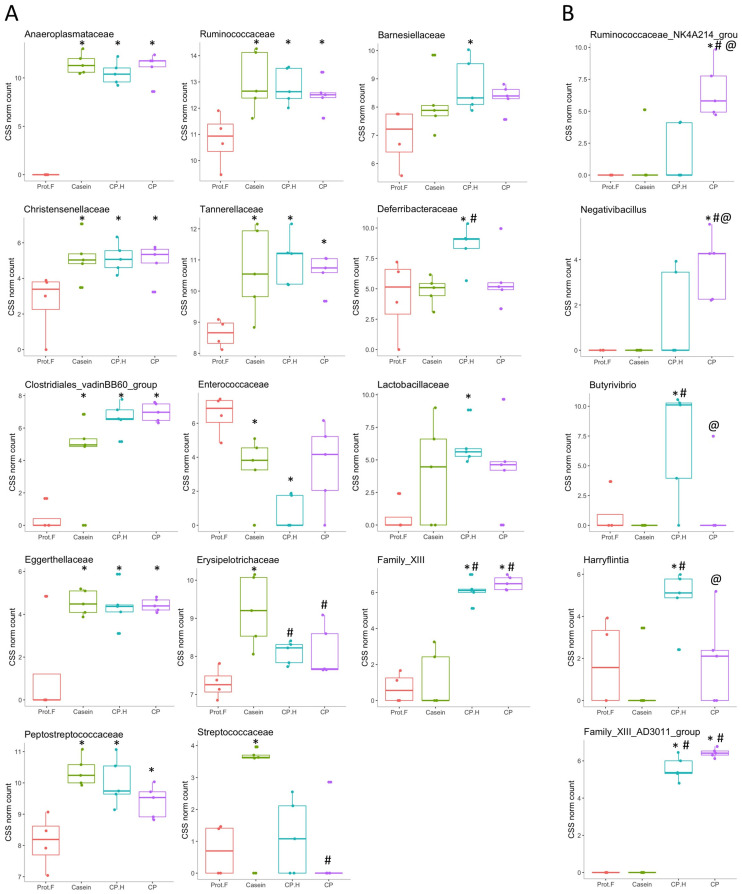
Assessment of differences in the family level (**A**) and in the genus level (**B**) of the gut microbiota of rats in receipt of different diets. * *p* < 0.1 denotes statistically different groups compared to the Prot-F group; # *p* < 0.1 denotes statistically different groups compared to the casein group. @ *p* < 0.1 denote statistically different groups compared to: cricket hydrolysates-enriched fermented beverage group. Prot.F: protein free-based control diet; Casein: casein-based control diet; CP.H: cricket hydrolysates-enriched fermented beverage; CP: whole-cricket powder-enriched fermented beverage.

At the family level (Figure 3B and Figure 4A), significant differences in abundance of several taxa were observed between groups fed with protein-based diets, independently of the protein type, and the group fed the protein free-based diet. Indeed, *Anaeroplasmataceae* (which belongs to the *Tenericutes* phylum) was only detected in the gut of animals fed with protein when compared to the group fed without protein, while *Christensenellaceae*, *Clostridiales_vadinBB60_group*, *Eggerthellaceae*, *Peptostreptococcaceae*, *Ruminococcaceae*, and *Tannerellaceae* were all increased in response to protein-supplemented diets (Figure 4A and Supplementary Table S1). Indeed, the increase in *Tenericutes* appears to be driven by the increase in the families of *Anaeroplasmataceae* and *Clostridiales_vadinBB60_group*. Interestingly, increased abundance of *Tenericutes*/*Anaeroplasmataceae* has been associated with positive energy balance [45], which is in line with our data, given that protein-fed rats were heavier than those on the protein-free diet. Based on these results, casein, fermented CP.H, and fermented CP could be defined as dietary components contributing to the maintenance of energy and supporting the growth in rodents. Furthermore, Yu et al. (2020) [46] reported that a duck egg-white diet led to a significant increase in the abundance of *Proteobacteria* and *Peptostreptococcaceae* in rats, supporting our data that the abundance of this family is increased with protein. In addition, *Peptostreptococcaceae* was suggested to maintain intestinal homeostasis in humans [46], suggesting that dietary protein can potentially contribute to intestinal health by supporting the abundance of this family.

Previous studies [42,47] showed that the abundance of *Ruminococcaceae* (with genes encoding for chitin-digesting enzymes) in the gut is thought to have roles in the fermentation of fibers. Thus, the increase of this family in the cricket-protein diets suggests that cricket chitin may be a source of fermentable and indigestible fiber, and that whole-cricket protein may favor the colonization of gut bacteria that have the capacity to degrade chitin. However, our results do not distinguish if chitin or protein led to that increase in *Ruminococcaceae*. Further targeted studies should be conducted to clarify this observation (Figure 3B). In addition, Nicholson et al. (2012) [48] showed that gut bacteria can utilize indigestible carbohydrates to produce short-chain fatty acids for colonocytes. Furthermore, short-chain fatty acids have been shown to be correlated with healthier metabolic states (e.g., better glucose homeostasis and lipid metabolism) and reduced colon-cancer risk [49]. These results confirmed that the fermented CP-based and CP.H-based beverages did not disrupt the healthy microbiota; on the contrary, they could be potential health-promoting ingredients. 

In contrast to the number of families that were observed to be increased similarly by all protein-supplemented diets, only *Enterococcaceae* was found to be decreased in a statistically significant manner, and then only by casein-fed and fermented CP.H-fed rats (though fermented CP-fed rats showed a tendency to have decreased levels as well) (Figure 4A). This family is one of the first to colonize the gut microbiota in response to exclusive breastfeeding and its abundance decreases gradually with the introduction of other foods [50]. Therefore, it is somewhat counterintuitive that we observed a decrease in response to protein feeding here; however, this is a rather broad family that includes many potential pathogens, many of which are of concern in the development of antibiotic resistance [51]. We were unable to detect changes in the abundance of genera belonging to this family; therefore, we cannot confirm the possibility that some potential pathogenic bacteria were decreased in response to our diets.

Our analysis also identified a subset of bacterial families whose abundances were specifically increased by individual protein-supplemented diets (Figure 4A). *Erysipelotrichaceae* and *Streptococcaceae* were increased by casein alone, with the former having higher abundances than both the fermented CP.H-supplemented diet and the CP-supplemented diet, while the latter had a significantly higher abundance than that of the CP-supplemented diet. *Streptococcaceae/Lactococcus* has been reported to be associated with inflammation and metabolic syndrome [52]. Other studies [53,54] found that the decrease in *Streptococcaceae/Lactococcus* might play an important role in the prevention of metabolic syndrome. In our study, the family of *Streptococcaceae* showed significantly lower levels in rats fed with CP compared to rats fed with casein, while *Lactococcus* abundance was lower for both fermented CP.H-based and CP-based diets compared to casein diets (*p* = 0.0028 and 0.0011, respectively; Supplementary Table S2).

In contrast, *Deferribacteraceae*, *Barnesiellaceae*, and *Lactobacillaceae* were only increased by CP.H [though trends for increases were observed in the CP-fed rats for the latter two as well). Similarly, Ijaz et al. (2020) [55] observed that mice fed with a pork-protein diet showed an increase in *Deferribacteraceae* and *Lactobacillaceae*. In contrast, Family_XIII was strongly increased with the cricket protein-based diets, independently of the protein form (hydrolysates or whole protein), but it was not altered by the casein diet. Family_XIII and *Clostridiales* (Figure 4A) may have roles in the metabolism of protein in the intestinal tract and are associated with the butyrate kinase butyrate–synthesis pathway that produces butyrate from protein [56,57]. Chai et al. (2019) [58] found that several genera within these families are potential butyrate producers, many of which are believed to have potential clinical applications, especially for the treatment of inflammatory bowel disease and *Clostridioides difficile* (*C. difficile*) infection [59].

When we went on to examine the effects of the different diets at the genus level, several interesting observations were made. *Anaeroplasma*, *Roseburia*, *Romboutsia*, and *Ruminiclostridium_9* were all significantly increased in all the protein-containing diets, compared to the protein-free control diet (Supplementary Table S2). These bacteria are associated with the fermentation of fiber into metabolites such as short-chain fatty acids, including acetate, propionate, and butyrate, which act as an anti-inflammatory constitutives [60]. In contrast, other genera showed protein-specific alterations in relative abundance. Rats fed with casein showed strong trends for increased abundance of UCG-014 and USG-005 (belonging to *Ruminococcaceae*), *Asteroleplasma*, and *Lactococcus* (though only the latter showed a statistically significant higher level, compared to every other diet), while *Ruminococcus_2* was decreased (Supplementary Table S2). Conversely, CP and/or CP.H specifically increased the abundances of *Parabacteroides*, *Tyzzerella*, *Intestinimonas*, and *Caproiciproducen,* associated with short-chain fatty-acid production [61], in comparison to the protein-free diet, and the abundances of *Anaerovorax*, the *Ruminococcaceae* genera UCG-007, UCG-009, UCG-013, and UBA1819 in comparison to the casein diet (Supplementary Table S2). This suggests that these latter genera are specifically responsive to the fermented protein diets and not just to protein-rich diets in general. The anti-inflammatory effect of *Parabacteroides goldsteinii* (belonging to *Parabacteroides*) has been investigated by Lai et al. (2022) [62], who reported that the bacteria could decrease the production of pro-inflammatory cytokines while increasing intestinal integrity. Interestingly, we identified several genera that were specifically increased by either CP (GCA-900066755, *Faecalitalea*, *Ruminococcaceae*_NK4A214_group, and *Negativibacillus*, with the latter two being significantly increased in CP vs. CP.H.; Figure 4B) or CP.H (GCA-900066575, *Candidatus_Soleaferrea*, *Butyrivibrio*, and *Harryflintia*, with the latter two being significantly increased in CP.H vs. CP (Figure 4B). These latter genera, therefore, appear to be very sensitive to whether the cricket protein is hydrolyzed or not. 

Family_XIII_AD3011_group was greatly increased in the fermented CP.H and CP groups compared to either control, where it was not detected, and thus it appears to be the driving force for the increased abundance of Family_XIII, mentioned above. Similarly, the *Lactobacillus* genus abundance mirrored that observed for *Lactobacillaceae*, being higher only in rats fed with CP.H compared to rats fed the protein-free diet, (*p* = 0.056; Supplementary data, Table S2), while the protein-free group had the lowest abundance of this genus. *Lactobacillus* spp. play key roles in the maintenance of metabolic balance [63] and in the reduction of the antigen load from gut microbiota to the host, resulting in an anti-inflammatory response [64]. In addition, they are important species within commercial probiotics [65,66,67]; thus, their increased abundance may confer a beneficial effect on animals fed the CP.H diet. The presence of *Lactobacillus* spp. in high abundance for rats fed with CP.H could be explained by the survival of *Lactobacillus* spp. from the fermented beverage during digestion. Indeed, the presence of proteins in the form of hydrolysates can be a more assimilable substrate for bacteria, which can influence the viability of these bacteria, potentially by increasing their ability to resist stressful gastrointestinal conditions. The CP-based diet did not have such an effect on the *Lactobacillus* spp. abundance, despite the presence of these bacteria in the fermented beverage. Thus, our results suggest that cricket-protein hydrolysates may be more beneficial for the protection and proliferation of *Lactobacillus* genera, compared to the whole-cricket protein.

### 3.2. Correlation between Bacterial Taxa and Nutritional Parameters 

The nutrition parameters related to growth, food, and protein intake, PER, TD, and AD, respectively, of the different diets (CP, CP.H, casein, and protein free-based diets) were evaluated in the previous study of [24]. Briefly, the results pointed out that the incorporation of CP.H, in addition to in vivo digestibility enhancement, increased the PER and the net protein ratio (NPR) significantly (*p* ≤ 0.05), compared to the incorporation of CP, from 1.7 to 2.0 and from 0.4 to 1.0, respectively. The AD of CP.H was 94%, which was close to that of the casein group (96%) and significantly (*p* ≤ 0.05) higher than that of the CP group (85%). 

**Figure 5:**
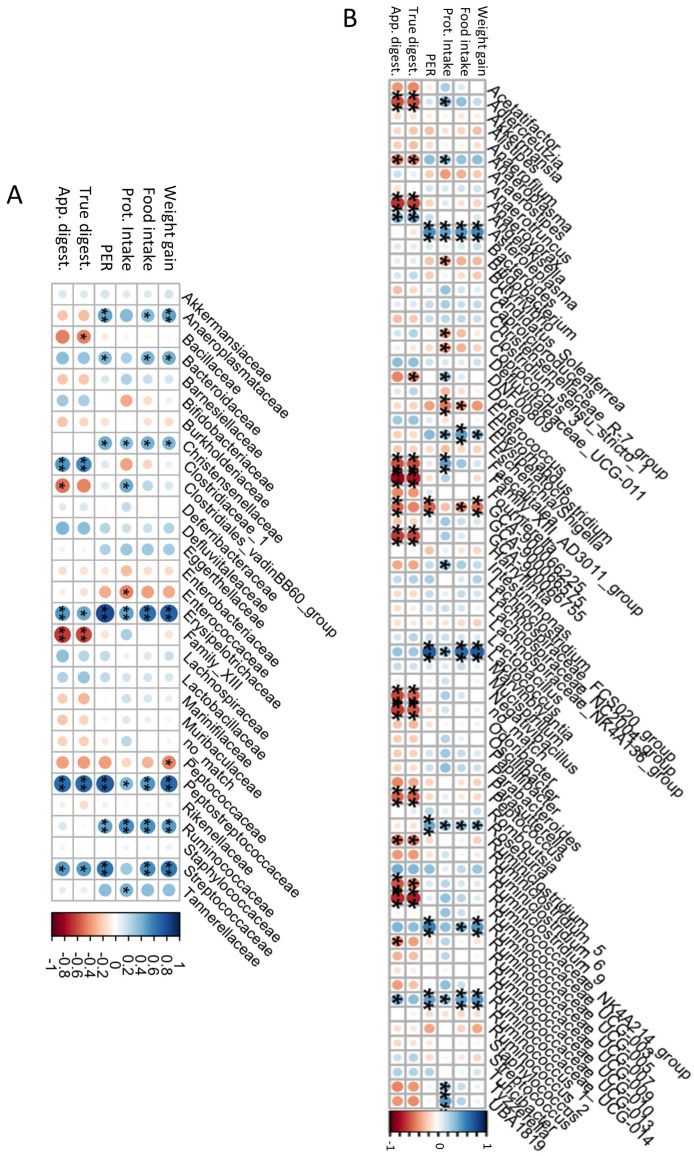
Associations among bacterial families (**A**), genera (**B**), and the nutritional quality. Prot: Intake: protein intake; PER: protein efficiency ratio; true digest: true digestibility; app. digest: apparent digestibility; * *p* < 0.1, ** *p* < 0.05.

The association of gut microbiota with the nutritional parameters was determined and the results are shown in Figure 5. At the family level (Figure 5A), *Anaeroplasmataceae*, *Bacteroidaceae, Christensenellaceae,* and *Ruminococcaceae* were positively correlated with weight gain (rho = 0.47, 0.42, 0.40, and 0.50, respectively), food intake (rho = 0.39, 0.41, 0.42, and 0.53, respectively), protein intake (rho = 0.38, 0.27, 0.38, and 0.54, respectively), and PER (rho = 0.47, 0.39, 0.43, and 0.47, respectively). The high abundance of these families reflects an increase in proteolytic bacteria with that of protein intake, and casein-based, CP-based, and CP.H-based diets did not produce differences in their abundances. *Erysipelotrichaceae, Peptostreptococcaceae*, and *Streptococcaceae* were positively correlated with all the nutritional parameters, as well as with digestibility (rho = 0.45, 0.68, and 0.50, respectively), with the latter having a statistically insignificant correlation with protein intake. *Clostridiales*_vadinBB60_group showed a positive correlation with protein intake (rho = 0.43) and a negative correlation with apparent digestibility (rho = −0.43), while *Enterococcaceae* was also negatively correlated with protein intake (rho = −0.43). Conversely, Family_XIII, which was particularly changed by the fermented CP.H-based and CP-based diets, showed a decrease when the digestibility increased (rho = −0.60). Similar negative correlations were observed for *Bacillaceae* and *Clostridiales*_vadinBB60_group with digestibility (rho = −0.44 and 0.43, respectively). Other bacteria did not show any correlation with nutritional parameters. Pozuelo et al. (2015) [68] observed a high proportion of *Erysipelotrichaceae* in healthy and lean individuals, related to the availability of butyrate. This suggests that a high-protein diet increases the abundance of *Erysipelotrichaceae* (high abundance observed for CP-based, CP.H-bsed, and casein-based diets), which may increase the availability of butyrate.

A study in humans [69] showed an anti-inflammatory property of butyrate, which could be implicated in the regulation of immune responses. In addition, butyrate has been shown to be effective in suppressing cancer and treating mucosal inflammation in both human and animal models [70,71]. Furthermore, Van der Wielen et al. (2000) [72] reported that the increase in butyric acid could be related to the decrease in the amount of *Enterobacteriaceae*. The positive correlation between *Ruminococcaceae* and the nutritional parameters suggests the importance of a protein-enriched diet for the protection of the intestinal microbiota. On the other hand, no correlation was observed between *Lactobacillaceae* abundance and the nutritional parameters mentioned above, despite the presence of these bacteria in the fermented CP.H-based and CP-based diets. These results support the specificity of the effects of a protein-based diet on specific bacterial taxa, although the influence of the included probiotics within the fermented beverage on the metabolic activities of the gut microbiota should be taken into account and are beyond the reach of this study. 

At the genus level (Figure 5B), we detected a dichotomy between genera with respect to their correlations with various parameters: while *Asteroleplasma*, *Erysipelatoclostridium*, *Lactococcus*, *Roseburia*, and the *Ruminococcaceae* genera *UCG-005* and *UCG-014* were all positively correlated with weight gain, food intake, protein intake, and PER, but not with digestibility, *Aldecreutzia*, *Anaerovorax*, *Faecalitalea*, Family_XII_AD3011_group, *GCA900066755*, *Negativibacillus*, *Peptococcus*, *Ruminoclostridium_9*, and *Ruminococcaceae_NK4A214_group* were all negatively correlated with digestibility intake (rho = −0.58, −0.25, −0.59, −0.75, −0.43, −0.53, −0.54, −0.50, and −0.73, respectively). *Bifidobacterium* was negatively correlated with protein intake (rho = −0.39). According to Stull et al. (2018) [15], a cricket-based diet could increase *Bifidobacterium* in humans. However, in our study, the abundance did not differ among diets. Similar observations were observed by Jarett et al. (2019) [42], who found that a cricket-based diet did not affect this genus in the gut microbiota of dogs. The presence of chitin, characterized by a structure similar to that of cellulose [73], could explain the high abundance of *Ruminococcaceae_NK4A214_group* in rats fed with the CP-based diet, as well as the negative correlation with the digestibility, as the CP showed low digestibility compared with casein and CP.H. *Erysipelatoclostridium*, considered to be a potential opportunistic pathogen [74,75], was observed to be lower for animals fed with fermented CP.H and CP, compared to those fed with a casein-diet or a protein free-based diet (see above). These results confirm that whole-cricket protein or cricket-protein hydrolysates may maintain a more balanced composition and reduce the pathogens within the gut bacteria. Other potential pathogens, such as those belonging to *Enterococcaceae*, showed a decrease when the weight gain, food intake, protein intake, and digestibility increased (rho = −0.38, −0.39, −0.45, and −0.35, respectively), indicating the importance of a diet rich in protein for the protection of the gut microbiota against pathogens.

## 4. Conclusions

This metagenomic study examined the impact of CP (whole-cricket protein)-enriched and CP.H (cricket protein hydrolysates)-enriched probiotic beverages on the gut microbiota of rats. The results of PCA and PCoA analyses showed that the global gut microbiota profile was modified by the diets containing fermented CP and CP.H proteins. While alpha-diversity was similarly increased by the fermented beverage diets and several bacterial taxa were identified as being similarly altered, independent of the proteins added (casein, fermented CP, or fermented CP.H), we did identify taxa that were either similarly or differentially affected by fermented CP-enriched and CP.H-enriched diets in comparison to the casein-enriched diet. These alterations likely resulted in the significant differences in overall gut microbiota architectures induced by the fermented CP-enriched and CP.H-enriched diets compared to the control diets. Moreover, the fermented CP.H-enriched probiotic beverage could be related to the decrease of the number of potential pathogenic members of *Enterococcaceae*. Correlation analysis highlighted the contribution of protein-enriched, fermented diets in general between nutritional parameters and the bacterial families *Anaeroplasmataceae*, *Christensenellaceae*, *Peptostreptococcaceae*, and *Ruminococcaceae*, all of which were similarly increased in these diets. At the genus level, *Asteroleplasma*, *Erysipelatoclostridium*, *Lactococcus*, and *Roseburia*, were all positively correlated with weight gain, food intake, protein intake, and PER, though these taxa were not altered by the protein-enriched fermented diets. Hence, these results suggest that protein-enriched, fermented diets alter the microbiome, in correlation with improved nutritional parameters, and that CP or CP.H may be viable alternative protein sources of probiotic product supplementation. It must be stated, however, that the above-mentioned effects cannot be separated from the general effects of the fermented probiotic beverage through this set of experiments. Therefore, further studies should be carried out to acquire a broader understanding of the impact of cricket protein-enrichment of fermented probiotic formulations on producing functional foods with effects on the gut microbiota and the ability to support digestive health.

## Data Availability

The original contributions presented in the study are included in the article/Supplementary Material, further inquiries can be directed to the corresponding author.

## Supplementary Materials

The following supporting information can be downloaded at: https://www.mdpi.com/article/10.3390/foods13020204/s1, Table S1: Assessment of differences in the family level of the gut microbiota of rats in receipt of different diets: *p*-value results; Table S2: Assessment of differences in the genera level of the gut microbiota of rats in receipt of different diets: *p*-value results.
