# Supplementary material for: Effect of a Probiotic Beverage Enriched with Cricket Proteins on the Gut Microbiota: Composition of Gut and Correlation with Nutritional Parameters
Source: Foods. 2024 Jan 9;13(2):204. doi: 10.3390/foods13020204 (PMC10814958; doi:10.3390/foods13020204)
Supplement: Supplementary file 1 [file foods-13-00204-s001.zip › foods-2731979-supplementary.pdf]

List of supplementary data

Table S1. Assessment of differences in the family level of the gut microbiota of rats in receipt of different diets: *P*-value results.

Table S2. Assessment of differences in the genera level of the gut microbiota of rats in receipt of different diets: *P*-value results.

Table S1.

Family	p Group	Casein-Prot.F	CP.H-Prot.F	CP-Prot.F	CP.H-Casein	CP-Casein	CP-CP.H
Anaeroplasmataceae	3.5e-10	9e-10	3.2e-09	1.4e-09	ns	ns	ns
Peptostreptococcaceae	0,00084	0,00074	0,0032	0,05	ns	ns	ns
Eggerthellaceae	0,0033	0,0058	0,0086	0,0076	ns	ns	ns
Ruminococcaceae	0,01	0,011	0,02	0,057	ns	ns	ns
Tannerellaceae	0,0072	0,022	0,0073	0,025	ns	ns	ns
Christensenellaceae	0,029	0,043	0,044	0,066	ns	ns	ns
Clostridiales_vadinBB60_group	3.8e-05	0,0062	9.6e-05	5.3e-05	ns	0,07	ns
Enterococcaceae	0,0026	0,083	0,0013	ns	ns	ns	ns
Deferribacteraceae	0,048	ns	0,06	ns	0,081	ns	ns
Erysipelotrichaceae	0,0039	0,0023	ns	ns	0,066	0,075	ns
Streptococcaceae	0,039	0,084	ns	ns	ns	0,045	ns
Family_XIII	7e-08	ns	3.6e-06	1.4e-06	5e-06	1.8e-06	ns
Barnesiellaceae	0,053	ns	0,039	ns	ns	ns	ns
Lactobacillaceae	0,076	ns	0,055	ns	ns	ns	ns
Akkermansiaceae	ns	ns	ns	ns	ns	ns	ns
Bacillaceae	ns	ns	ns	ns	ns	ns	ns
Bacteroidaceae	ns	ns	ns	ns	ns	ns	ns
Bifidobacteriaceae	ns	ns	ns	ns	ns	ns	ns
Burkholderiaceae	ns	ns	ns	ns	ns	ns	ns
Clostridiaceae_1	ns	ns	ns	ns	ns	ns	ns
Defluviitaleaceae	ns	ns	ns	ns	ns	ns	ns
Enterobacteriaceae	ns	ns	ns	ns	ns	ns	ns
Lachnospiraceae	ns	ns	ns	ns	ns	ns	ns
Marinifilaceae	ns	ns	ns	ns	ns	ns	ns
Muribaculaceae	ns	ns	ns	ns	ns	ns	ns
no_match	ns	ns	ns	ns	ns	ns	ns
Peptococcaceae	ns	ns	ns	ns	ns	ns	ns

Rikenellaceae	ns	ns	ns	ns	ns	ns	ns
Staphylococcaceae	ns	ns	ns	ns	ns	ns	ns

Prot.F, Protein free-based diet; Casein, casein-based control diet; CP.H, Cricket hydrolysates enriched fermented beverage; [2]CP, Whole cricket powder enriched fermented beverage.

Table S2.

Genus	p Group	Casein-Prot.F	CP.H-Prot.F	CP-Prot.F	CP.H-Casein	CP-Casein	CP-CP.H
Anaeroplasma	7.6e-10	3.8e-09	5.6e-09	1.9e-09	ns	ns	ns
Roseburia	0,0029	0,0029	0,018	0,0078	ns	ns	ns
Romboutsia	0,014	0,039	0,013	0,049	ns	ns	ns
Ruminiclostridium_9	0,00047	0,048	0,0028	0,00037	ns	0,073	ns
Enterococcus	0,004	0,067	0,0022	ns	ns	ns	ns
Ruminococcaceae_UCG-014	0,056	0,059	ns	ns	ns	ns	ns
Ruminococcaceae_UCG-005	0,071	0,068	ns	ns	ns	ns	ns
Asteroleplasma	0,069	0,074	ns	ns	ns	ns	ns
Lactococcus	5e-04	0,0019	ns	ns	0,0028	0,0011	ns
Ruminococcus_2	0,015	0,058	ns	ns	0,013	0,091	ns
Anaerovorax	0,0013	ns	0,081	0,0042	0,068	0,0029	ns
Ruminococcaceae_UCG-007	3.2e-10	ns	1.7e-08	3.4e-08	7.4e-09	1.5e-08	ns
Ruminococcaceae_UCG-009	0,00023	ns	0,00054	0,0051	0,0026	0,029	ns
Ruminococcaceae_UCG-013	2.6e-05	ns	2e-04	0,00039	0,00061	0,0013	ns
UBA1819	0,0051	ns	0,036	0,069	0,017	0,034	ns
Papillibacter	0,0051	ns	0,0048	0,047	0,052	ns	ns
Parabacteroides	0,0057	ns	0,0069	0,011	ns	ns	ns
Tyzzerella	0,0049	ns	0,007	0,011	ns	ns	ns
Intestinimonas	0,054	ns	0,064	0,069	ns	ns	ns
Caproiciproducens	0,034	ns	0,041	0,1	ns	ns	ns
Christensenellaceae_R-7_group	0,075	ns	0,083	0,1	ns	ns	ns
Parasutterella	0,051	ns	ns	ns	0,07	0,084	ns
Anaerostipes	0,0052	ns	0,0061	0,061	0,033	ns	ns
Erysipelatoclostridium	0,052	ns	0,07	0,066	ns	ns	ns
GCA-900066575	0,07	ns	0,047	ns	ns	ns	ns
Lactobacillus	0,07	ns	0,056	ns	ns	ns	ns

DNF00809	0,036	ns	0,062	ns	ns	ns	ns
Oscillibacter	0,085	ns	0,071	ns	ns	ns	ns
Candidatus_Soleaferrea	0,0053	ns	0,0053	ns	0,026	ns	ns
Mucispirillum	0,057	ns	0,1	ns	0,064	ns	ns
Family_XIII_AD3011_group	5.9e-15	ns	1.5e-12	0	1.7e-13	0	0,0095
Butyrivibrio	0,013	ns	0,049	ns	0,014	ns	0,061
Harryflintia	0,015	ns	0,094	ns	0,012	ns	0,093
Adlercreutzia	0,081	ns	ns	0,084	ns	ns	ns
GCA-900066755	0,0023	ns	ns	0,0059	ns	0,0037	ns
Faecalitalea	0,029	ns	ns	0,032	ns	0,084	ns
no_match	0,03	ns	ns	0,1	ns	0,074	ns
Ruminococcaceae_NK4A214_group	0,00062	ns	ns	0,00095	ns	0,0025	0,0067
Negativibacillus	0,0012	ns	ns	0,0032	ns	0,0019	0,064
Ruminiclostridium_5	0,1	ns	ns	ns	ns	ns	ns
Anaerovorax	0,0013	ns	0,081	0,0042	0,068	0,0029	ns
Ruminococcaceae_UCG-007	3.2e-10	ns	1.7e-08	3.4e-08	7.4e-09	1.5e-08	ns
Ruminococcaceae_UCG-009	0,00023	ns	0,00054	0,0051	0,0026	0,029	ns
Ruminococcaceae_UCG-013	2.6e-05	ns	2e-04	0,00039	0,00061	0,0013	ns
UBA1819	0,0051	ns	0,036	0,069	0,017	0,034	ns
Papillibacter	0,0051	ns	0,0048	0,047	0,052	ns	ns
Parabacteroides	0,0057	ns	0,0069	0,011	ns	ns	ns
Tyzzerella	0,0049	ns	0,007	0,011	ns	ns	ns
Intestinimonas	0,054	ns	0,064	0,069	ns	ns	ns
Caproiciproducens	0,034	ns	0,041	0,1	ns	ns	ns
Christensenellaceae_R-7_group	0,075	ns	0,083	0,1	ns	ns	ns
Parasutterella	0,051	ns	ns	ns	0,07	0,084	ns
Anaerostipes	0,0052	ns	0,0061	0,061	0,033	ns	ns
Erysipelatoclostridium	0,052	ns	0,07	0,066	ns	ns	ns
GCA-900066575	0,07	ns	0,047	ns	ns	ns	ns
Lactobacillus	0,07	ns	0,056	ns	ns	ns	ns

DNF00809	0,036	ns	0,062	ns	ns	ns	ns
Oscillibacter	0,085	ns	0,071	ns	ns	ns	ns
Candidatus_Soleaferrea	0,0053	ns	0,0053	ns	0,026	ns	ns
Mucispirillum	0,057	ns	0,1	ns	0,064	ns	ns
Family_XIII_AD3011_group	5.9e-15	ns	1.5e-12	0	1.7e-13	0	0,0095
Butyrivibrio	0,013	ns	0,049	ns	0,014	ns	0,061
Harryflintia	0,015	ns	0,094	ns	0,012	ns	0,093
Adlercreutzia	0,081	ns	ns	0,084	ns	ns	ns
GCA-900066755	0,0023	ns	ns	0,0059	ns	0,0037	ns
Faecalitalea	0,029	ns	ns	0,032	ns	0,084	ns
no_match	0,03	ns	ns	0,1	ns	0,074	ns
Ruminococcaceae_NK4A214_group	0,00062	ns	ns	0,00095	ns	0,0025	0,0067
Negativibacillus	0,0012	ns	ns	0,0032	ns	0,0019	0,064

Prot.F, Protein free-based diet; Casein, casein-based control diet; CP.H, Cricket hydrolysates enriched fermented beverage; [2]CP, Whole cricket powder enriched fermented beverage.
